# Orexinergic modulation of chronic jet lag-induced deficits in mouse cognitive flexibility

**DOI:** 10.1038/s41386-024-02017-8

**Published:** 2024-10-30

**Authors:** Julius Duske, Nicole D’Souza, Dana Mayer, Daniela C. Dieterich, Markus Fendt

**Affiliations:** 1 Institute for Pharmacology and Toxicology, Medical Faculty, Otto-von-Guericke University, Magdeburg, Germany; 2 Center of Behavioural Brain Sciences, Otto-von-Guericke University, Magdeburg, Germany; 3 Present Address: Institute of Neurophysiology, Goethe University, Frankfurt, Germany

**Keywords:** Hypocretin, Working memory, Cortex

## Abstract

Cognitive flexibility and working memory are important executive functions mediated by the prefrontal cortex and can be impaired by circadian rhythm disturbances such as chronic jet lag (CJL) or shift work. In the present study, we used mice to investigate whether (1) simulated CJL impairs cognitive flexibility, (2) the orexin system is involved in such impairment, and (3) nasal administration of orexin A is able to reverse CJL-induced deficits in cognitive flexibility and working memory. Mice were exposed to either standard light-dark conditions or simulated CJL consisting of series of advance time shifts. Experiment (1) investigated the effects of a mild CJL protocol on cognitive flexibility using the attentional set shifting task. Experiment (2) used a stronger CJL protocol and examined CJL effects on the orexin system utilizing c-Fos and orexin immunohistochemistry. Experiment (3) tested whether nasal orexin application can rescue CJL-induced deficits in cognitive flexibility and working memory, the latter by measuring spontaneous alternation in the Y-maze. The present data show that CJL (1) impairs cognitive flexibility and (2) reduces the activity of orexin neurons in the lateral hypothalamus. (3) Nasal administration of orexin A rescued CJL-induced deficits in working memory and cognitive flexibility. These findings suggest that executive function impairments by circadian rhythm disturbances such as CJL are caused by dysregulation of orexinergic input to the prefrontal cortex. Compensation of decreased orexinergic input by nasal administration of orexin A could be a potential therapy for CJL- or shift work-induced human deficits in executive functions.

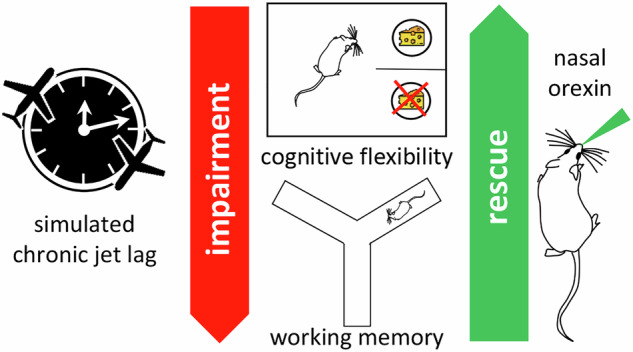

## Introduction

Circadian rhythm sleep disorder (CRSD) is a disorder that affects the timing of sleep. It is caused by a mismatch of the endogenous clock and externally set stimuli such as light or dark outdoors, physical activity, or meal times. CRSD is either intrinsic such as advanced or delayed sleep-wake disorder or extrinsic including shift work disease or jet lag, which is more common. For both variants, the main clinical manifestations are insomnia and excessive daytime sleepiness [1]. Of interest, cognitive impairments and associated changes in the brain can be observed in CRSD. For example, chronic jet lag (CJL) in flight attendants has been associated with cognitive deficits, particularly in working memory, as well as atrophy of the temporal lobe [2, 3]. In addition, impairments in attention and decision-making due to chronic sleep deprivation have been demonstrated [4]. Although chronotherapy, photic treatment, and cognitive-behavioural therapy exist, pharmacological treatment for CRSD is mainly limited to melatonin [5].

Cognitive impairments were also observed in laboratory rodents exposed to simulated CJL. Reduce hippocampal neurogenesis was found after phase shortening (simulating travelling east), which was associated with impaired spatial learning [6]. Other forms of circadian desynchronization (e.g. transferring the animals to a 10:10 h light/dark cycle or to a dark/dark cycle) have been shown to affect the transcriptional profile and the physiological rhythms of neurons in the prefrontal cortex, leading to emotional changes and cognitive deficits [7–10]. However, there is poor knowledge about the effects of simulated CJL on executive functions in laboratory rodents. Executive functions include working memory, inhibitory control, attention, and cognitive flexibility [11] and can be measured in mice using specific behavioural paradigms [12]. A well-established paradigm to measure cognitive flexibility is the attentional set shifting task (ASST) [13, 14]. The ASST is based on discrimination learning and includes different types of transfers such as reversals, intra- and extradimensional shifts. Working memory can be measured by spontaneous alternations in the Y-maze [15, 16].

The aim of the present study is to investigate whether the orexin system in mice is involved in cognitive deficits induced by simulated CJL. The orexin system consists of neurons in the lateral hypothalamus that synthesize the neuropeptides orexin A and B and their brain-wide projections [17–19]. This system is involved in a variety of regulatory mechanisms including stress responses, emotions, motivation, eating behaviour and sleep-wake cycle [20–22]. The orexin system supports arousal, for example through projections into wake-promoting brain regions such as the locus coeruleus and the tuberomammilary nucleus [23]. Studies first in animal models [24–26] and later in humans [27, 28] highlighted the importance of the orexin system in narcolepsy. This disease with symptoms such as excessive daytime sleepiness and sleep attacks is a consequence of loss of orexin neurons or disturbed orexin receptor signalling. This suggests that orexin or orexin receptor agonists could be a promising pharmacological treatment option for CJL-induced symptoms as observed in CRSD. Such a treatment would not only address symptoms associated with disturbed sleep-wake cycle but potentially also cognitive symptoms because the orexin system also plays a role in cognition. In laboratory animals, orexin administration rescued impaired discrimination learning [29] and improved executive functions such as cognitive flexibility or attention [30].

Thus, in the present study, we exposed male and female mice to simulated CJL, an animal model of CRSD. Then, we investigated whether (1) simulated CJL impairs cognitive flexibility, (2) the orexin system is involved in such an impairment, and if so, whether (3) nasal administration of orexin A is able to reverse CJL-induced deficits in cognitive flexibility and working memory. Our hypothesis is that (1) simulated CJL induces impairments in cognitive flexibility and working memory, (2) these impairments are associated with changes in the orexin system, and (3) nasal administration of orexin rescues CJL-induced cognitive deficits.

## Materials and methods

### Animals

Female and male C57BL/6 J mice were used in the experiments. Mice were housed in groups of up to 8 mice/cage under controlled conditions (humidity: 55 ± 10%, temperature: 22 ± 2 °C) and – depending on the group – either in a standard 12 h light/12 h dark cycle (light on: 6:00 am) or in a CJL cycle (see below). Mice were between 12 and 15 weeks of age during testing. Food and water were provided *ad libitum* until 1 week before and during ASST when food was restricted (approximately 2.5 g/mice/day) to maintain 90–95% of the mice’s basal body weight. All experiments were complied with the International Guidelines for the Care and Use of Animals for Experimental Procedures (2010/63/EU) with confirmed approval from the local authorities (Landesverwaltungsamt Sachsen-Anhalt, Az. 42502-2-1618 UniMD).

Of note, all behavioural experiments were performed during the light period (brightness in the setups: 100–200 lx) and started approx. 8:00–9:00 am. The brains for experiment 2 (immunohistochemistry) were collected one hour later.

### Simulation of chronic jet lag (CJL)

**Fig. 1:**
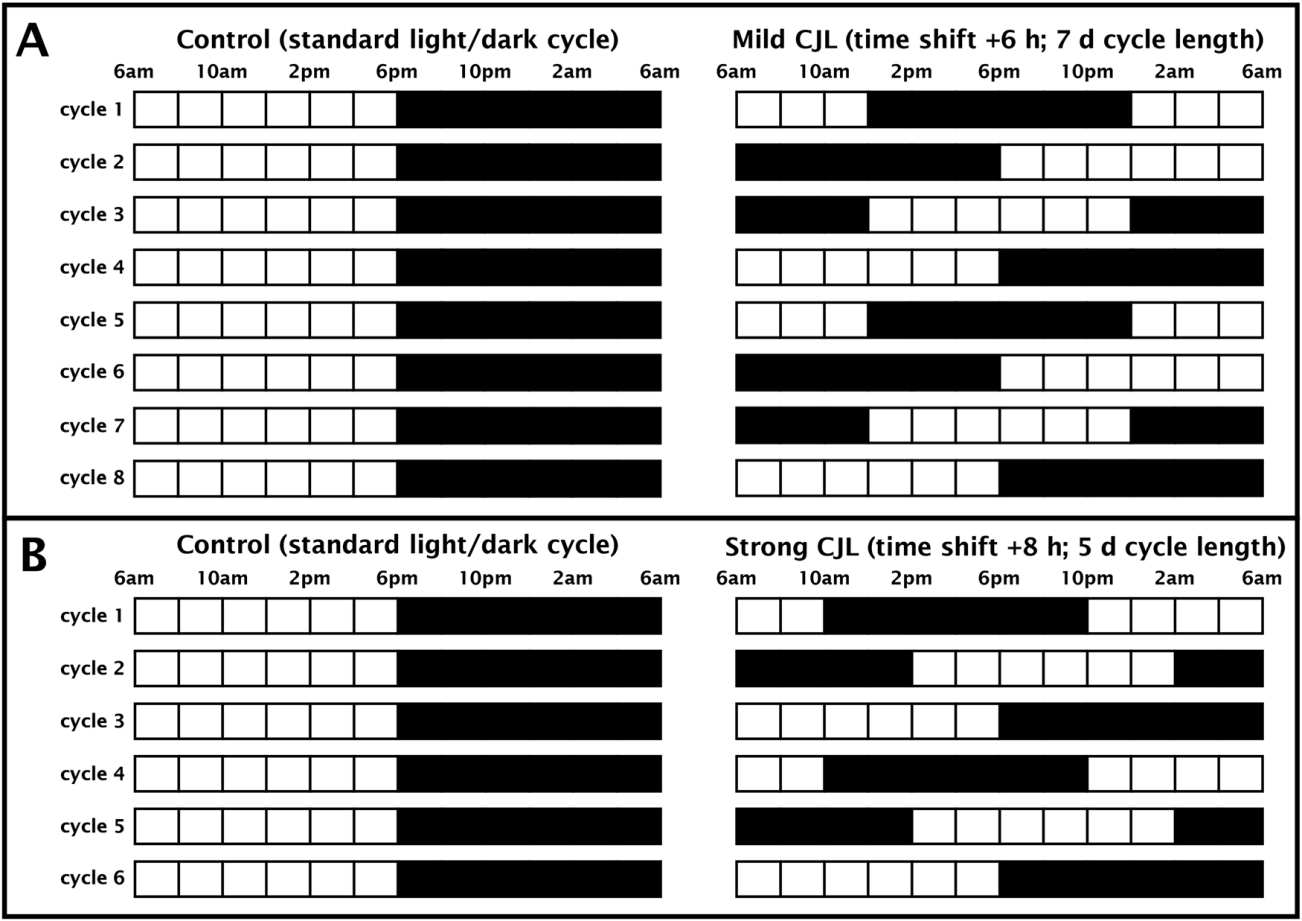
Light-dark cycles of the CJL protocols. **A** The mild CJL protocol consisted of 6 h-shifts every 7 days. One experimental group was tested after 4-time shifts (i.e., in cycle 4), and the other group after 8-time shifts (i.e., in cycle 8). **B** The strong CJL protocol consisted of 8 h-shifts every 5 days and the experiments were performed after 6-time shifts (i.e., in cycle 6). CJL chronic jet lag, control standard light-dark cycle.

After an adaptation period of at least 1 week, CJL was simulated for the experimental groups by phase advancing of the light-dark cycle, while the control groups stayed in the standard light-dark cycle. In experiment 1, the light-dark conditions were shifted by 6 h every seventh day (mild CJL protocol; Fig. 1A). Behavioural tests were performed after 4 or 8-time shifts, respectively (i.e., in week 4 or 8 after starting CJL). In experiments 2 and 3, the light-dark conditions were shifted by 8 h every fifth day (strong CJL protocol; Fig. 1B). Behavioural tests or immunohistochemistry, respectively, were performed after 6-time shifts, i.e., in week 4 after starting CJL.

### Behavioural experiments

#### Attentional set shifting task (ASST)

The ASST was performed as previously described [29, 31, 32]. For detailed information please see supplementary information.

Briefly, custom-made boxes were used consisting of a waiting and two choice compartments. During habituation, mice were familiarized with bowls and rewards, were handled daily for a week, and subjected to food restriction. A pre-training involved digging in bowls filled with bedding material to find rewards. For experiment 3, mice also underwent nasal saline application habituation.

The testing phase began immediately after habituation, lasting 4 days. Bowls were presented with visual-tactile cues (digging media) or olfactory cues (odorants), with only one containing a reward. Mice had to associate the correct cue with the reward, with bowl positions randomized. Trials started with a 30-s wait period, and then sliding doors to the choice compartments were opened. Correct choices allowed mice to consume the reward, while incorrect choices were indicated by removing the empty bowl. After six consecutive correct decisions, a phase was considered to be completed and the next phase started. As dependent variables, the number of trials and errors until completion was used.

The ASST had several phases:

- Day 1; Simple Discrimination (SD): Mice chose between two exemplars of one stimulus dimension (odor or digging medium).
- Day 2; Compound Discrimination (CD): An additional, irrelevant stimulus dimension was introduced, followed by a reversal phase (Rev1) where contingencies changed.
- Day 3; Intradimensional Shift (IDS): New exemplars of both dimensions were presented, with the relevant dimension of the previous phases predicting the reward, followed by another reversal (Rev2).
- Day 4; Extradimensional Shift (EDS): New exemplars of both dimensions were used, with the previously irrelevant dimension now predicting the reward, followed by a final reversal phase (Rev3).

#### Spontaneous alternation in the Y-maze

The custom-made Y-maze consisted of 3 arms, 120 degrees apart, 37 cm long, 10 cm wide, and 13 cm high. The walls were transparent, the floor of grey PVC, and the arms were not baited. The experiment started with placing the mice in one arm of the maze, facing to the centre. Then, the mouse could freely explore the Y-maze for 5 min. The experimenter manually scored arm entries of the mice, defined by entering an arm with all four paws. The percentage of successful alternations, i.e., (number of successful alternations)/(total arm entries – 2) × 100, and arm entries were used as independent variables. Successful alternations were defined as three consecutive visits of all three arms of the Y-maze without repetitions. Since spontaneous alternations are considerably above the chance level (50%), it is assumed that the mice remember which arm was last entered and that they enter the least new arm due to their preference for novelty – a process that involves working memory [15, 16]. Spontaneous alternations at chance level or significantly lower than in the control group indicate poor or deficient working memory.

### Immunohistochemistry

Mice were deeply anaesthetized with i.p. administration of ketamine hydrochloride (100 mg/kg), xylazine hydrochloride (20 mg/kg), and acepromazine maleate (3 mg/kg) and transcardially perfused, first with saline (2 min), then with Zamboni solution (4% paraformaldehyde, 0.2% picric acid in PBS; 10 min). The brain was removed, post-fixed overnight, and then stored in 30% sucrose. Using a cryostat, coronal 40 µm sections were cut.

Double-immunofluorescence staining was performed on every third section of the regions of interest (lateral hypothalamus, prefrontal cortex). For c-Fos staining, a rabbit anti-c-Fos antibody (1:3000, #226008, Synaptic Systems GmbH, Göttingen, Germany), a biotinylated goat anti-rabbit IgG antibody (1:1000, BA-1000-1.5, Vector Laboratories, Newark, USA), and an ABC kit (PK-6100, Vector Laboratories, Newark, USA) were used. For orexin A staining, a mouse anti-orexin A antibody (1:3000, sc-80263, Santa Cruz Biotechnology, Santa Cruz, CA, USA), Cy 3-conjugated goat anti-mouse IgG antibody (1:400, #115-165-166, Jackson ImmunoResearch, Laboratories, Inc., Ely, Cambridgeshire, United Kingdom), and Streptavidin Cy-2 (S11223, 1:500, ThermoFisher Scientific, Darmstadt, Germany) were used. The stained slides were mounted on microscope slides and coverslips were added. Photomicrographs were taken using a confocal laser scanning microscope (10× magnification; Axio observer.Z1 microscope provided with a LSM 710 and LSM 800 confocal system, Carl Zeiss AG, Oberkochen, Germany) with respective software.

For quantitative analysis of the photomicrographs, pre-defined frames (415 µm × 415 µm for the PFC regions, 600 µm × 500 µm for the lateral hypothalamus) were placed bilaterally in the centre of the lateral hypothalamus, ventromedial PFC, cingulate cortex, lateral/ventral OFC or medial OFC, respectively, with the help of coronal plates if a mouse brain atlas (see also Fig. 3). In the lateral hypothalamus, c-Fos-positive neurons, orexin A-positive neurons and double-labelled neurons (defined by clear signals of both dyes, c-Fos in the nucleus and orexin in the cytoplasm of the same neuron) were manually counted by the blinded experimenter. c-Fos-positive neurons of the different subregions of the PFC were counted with custom-made software (see supplementary information). Between 3 and 5 slides per hemisphere between the following anterior-posterior coordinates (from bregma) were analysed: lateral hypothalamus: from −1.2 mm to −1.8 mm; ventromedial PFC: from 1.9 mm to 1.5 mm; cingulate cortex: from 1.6 mm to 1.4 mm; OFC: from 2.80 mm to 2.10 mm. For every individual mouse, the average number of counted neurons per mm^2^ (including all counts from both hemispheres) was calculated and used for statistical analyses.

### Drugs

In experiment 3, mice were treated with either 10 μl vehicle (saline) or 10 μl orexin A solution (0.1 mM; Tocris Bioscience, Bristol, UK). Concentration and volume were based on published data [29, 30].

### Experimental procedure

#### Experiment 1

Mice were kept in the standard light/dark cycle (control) or in a mild CJL (see Fig. 1A). After 4 or 8-time shifts (i.e., at the beginning of cycle 4 or 8), respectively, mice were submitted to the ASST (approximately 8:00–9:00 am). Group sizes: control: 5 females, 6 males; 1-month CJL: 5 females, 6 males; 2 months CJL: 5 females, 6 males.

#### Experiment 2

Mice were kept in standard light/dark cycle (control) or strong CJL for 6 cycles (see Fig. 1B). One hour after the start of the behavioural tests in experiments 1 and 3 (i.e., 9:00–10:00 am), the mice were transcardially perfused. The brain was then removed and processed for immunohistochemistry as described. Group sizes: control: 7 females, 7 males; CJL: 7 females, 6 males.

#### Experiment 3

Mice were kept in standard light/dark cycle (control) or strong CJL (see Fig. 1B). After 6-time shifts (i.e. at the beginning of cycle 6), mice were submitted to the Y-maze test and 5 min later to ASST (approximately 8:00–9:00 am). On each of the four days with ASST, thirty minutes before the start of the Y-maze experiment, the mice were treated with nasal administrations of saline (vehicle) or orexin A. Of note, some mice were tested only in the Y-maze but not in the ASST (since throughput is limited in the latter but not in the former). However, all mice subjected to ASST were previously tested in the Y-maze. Group sizes (Y-maze/ASST): control/vehicle: 9/6 females, 9/6 males; CJL/vehicle: 7/6 females, 9/6 males; control/orexin A: 9/6 females, 8/6 males; CJL/orexin A: 7/6 females, 10/6 males.

### Statistical analysis

Data analysis was performed using SYSTAT 13 (Systat Software GmbH, Düsseldorf, Germany) and Prism 7.0 (GraphPad Software Inc., La Jolla, USA). Normal distribution of the data was verified using the Shapiro–Wilk normality test. Analysis of variance (ANOVA) was performed, followed by respective post-hoc multiple comparisons (two-linear step-up procedure of Benjamini, Krieger and Yekutieli). The significance level was set at *p* < 0.05.

## Results

### Experiment 1

A multi-factorial ANOVA using sex (female/male) and cycle condition (control, 1-month CJL, 2 months CJL) as between-subject factors and ASST phase (SD, CD, Rev1, IDS, Rev2, EDS, Rev3) as within-subject factor was used to analyse ASST performance of the mice.

**Fig. 2:**
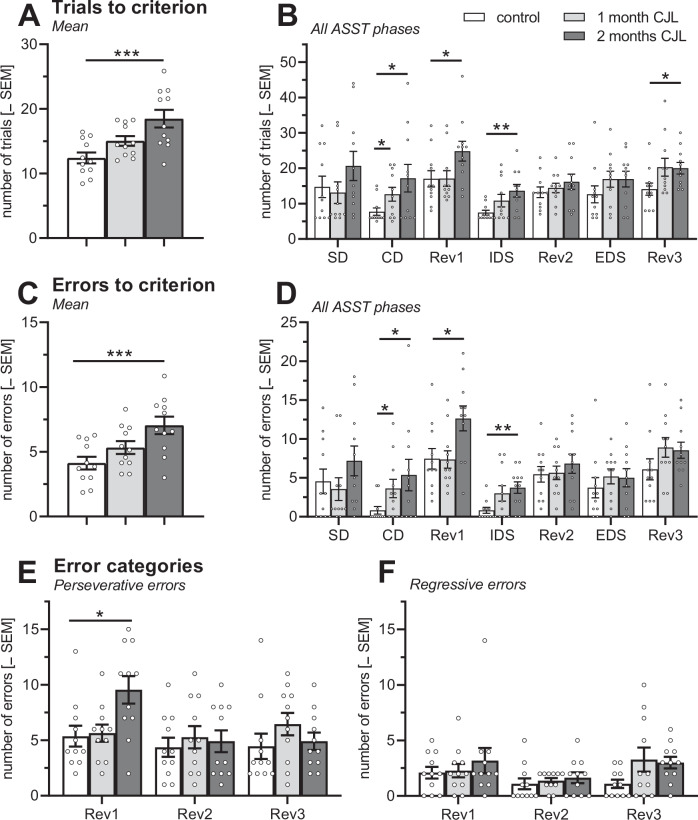
ASST performance after 1 or 2 months of simulated CJL. **A** Two but not one month of CJL increase the mean number of trials to criterion compared with the standard light/dark cycle. **B** This effect was most pronounced in the CD, Rev1, IDS and Rev3 phases of ASST. **C** Very similar effects were observed on the mean number of errors to criterion. **D** These effects were most pronounced in the CD, Rev1 and IDS phases. **E** Perseverative and **F** regressive errors were not affected on a statistical level. ***p < 0.001, **p < 0.01, *p < 0.05, post-hoc com*p*arisons (as indicated) after significant effects in ANOVA. The dots represent the individual measures. ASST attentional set shifting task, CD compound discrimination, CJL chronic jet lag, EDS extradimensional shift, IDS intradimensional shift, Rev1-3 reversal 1-3, control standard light-dark cycle.

Since this multi-factorial ANOVA showed neither main effects of sex (*F*s < 1.63; *p*s > 0.21) nor interactions of sex with other factors (*F*s < 2.17; *p*s > 0.13) for trials to criterion (Fig. 2A, B), errors to criterion (Fig. 2C, D), and the errors types (Fig. 2E, F), the data from both sexes were pooled for further analysis.

Regarding trials and error to criterion, the analysis of the pooled data revealed the main effects of cycle conditions (*F*s > 6.84, *p*s < 0.004) and ASST phase (*F*s > 5.49, *p*s < 0.004) but no interaction between these two factors (Fs < 1.00, *p*s > 0.45). Post-hoc comparisons showed that 1-month CJL did not induce a performance deficit (*q*s < 1.81, *p*s > 0.14) while 2 months CJL significantly impaired ASST performance (*q*s > 3.68, *p*s < 0.002; Fig. 2A, C). This effect of 2 months CJL was most robust in the CD, Rev1, IDS and Rev3 phases (*t*s > 2.16, *p*s < 0.05; Fig. 2B, D). Analysis of the error types in the reversal phases revealed a trend for interaction for the ASST phase and CJL on perseverative errors (*F*(4,60) = 2.45, *p* = 0.055; post-hoc comparison control vs. 1-month CJL in Rev1: *t* = 2.69, *p* = 0.02), as well as a main effect of phase (*F*(4,60) = 3.64, *p* = 0.04). There were no significant effects on regressive errors (*F*s < 2.74, *p*s > 0.08).

Separate analyses of female and male mice further revealed that the effects of CJL on ASST performance were more pronounced in female than in male mice (Figs. S1, S2).

### Experiment 2

**Fig. 3:**
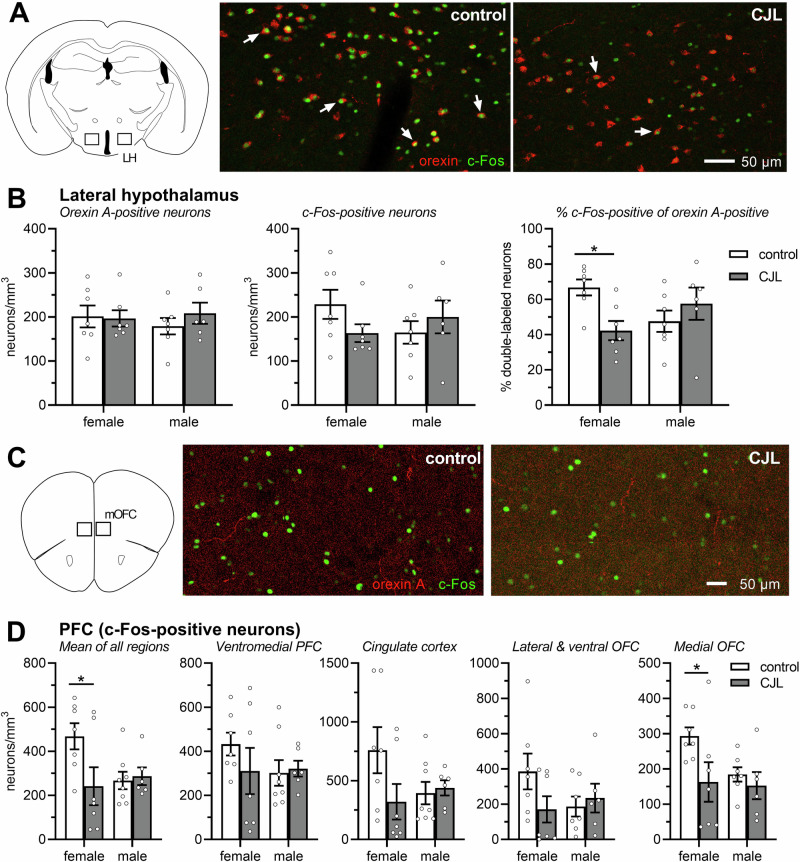
Effect of cycle condition and sex on c-Fos expression in orexin-positive neurons in the lateral hypothalamus and neurons in ASST-associated PFC regions. **A** depicts examples of orexin- and c-Fos-positive neurons in the lateral hypothalamus (LH) of mice exposed to the standard light-dark cycle (ST) or chronic jet lag (CJL). The arrows point to representative double-positive neurons. **B** Exposure to CJL as well as sex did not affect the number of orexin- and c-Fos-positive neurons. However, in female mice, less orexin-positive neurons were also c-Fos-positive when they were exposed to CJL. This suggests a decreased activity of orexin neurons after CJL exposure. **C** shows c-Fos-positive neurons in the medial orbitofrontal cortex (mOFC) after exposure to the control or CJL. Note the presence of orexin-positive fibres in the mOFC. **D** Very similar sex-specific effects were observed in the regions of the PFC that are associated with cognitive flexibility. In female but not male mice, exposure to CJL reduced the number of c-Fos-positive neurons. This effect was most pronounced in the medial OFC. *p < 0.05, post-hoc comparison (as indicated) after significant effects in ANOVA. The dots represent the individual measures. CJL simulated chronic jet lag, LH lateral hypothalamus, mOFC medial orbitofrontal cortex, OFC orbitofrontal cortex, PFC prefrontal cortex, control standard light-dark cycle.

The number of c-Fos- and orexin A-positive neurons, as well as the percentage of double-positive neurons (normalized to the number of orexin A-positive neurons) in the lateral hypothalamus, was analysed with a multi-factorial ANOVA using sex (female/male) and cycle condition (control, CJL) as between-subject factors. Figure 3A shows examples of immunohistochemical staining. Of note, a strong CJL protocol was used in this experiment.

Both, the number of orexin A- and c-Fos-positive neurons were not affected by sex or cycle condition (*F*s < 0.34, *p*s > 0.57; Fig. 3B). While these two factors did not interact regarding the number of orexin A-positive neurons (*F*(1,23) = 0.62, *p* = 0.44), there was a trend for such an interaction regarding the c-Fos-positive neurons (*F*(1,23) = 2.95, *p* = 0.099). Of note, this interaction between sex and cycle condition was significant in the analysis of the percentage of double-positive neurons (*F*(1,23) = 7.40, *p* = 0.01), whereas there were no main effects of sex and cycle condition (*F*s < 1.32, *p*s > 0.26). Post-hoc comparison revealed a significant decrease of double-positive neurons after CJL in female mice (*t* = 2.79, *p* = 0.01) but not in male mice (*t* = 1.09, *p* = 0.29).

In addition, c-Fos expression (Fig. 3C, D) was analysed in the regions of the PFC associated with cognitive flexibility (ventromedial PFC, cingulate cortex, lateral/ventral orbitofrontal cortex (OFC) and medial OFC). There were no main effects of sex and cycle condition in the different PFC regions (*F*s < 2.51, *p* > 0.12), except a main effect of cycle condition in the medial OFC (*F*(1,23) = 4.63, *p* = 0.04). Furthermore, there was again an interaction of sex and cycle condition when the number of c-Fos-positive neurons in all these PFC regions was averaged (*F*(1,24) = 4.24, *p* = 0.05; Fig. 3D). Post-hoc comparisons showed a decreased number of c-Fos-positive neurons in female mice after CJL exposure in both the medial OFC and the mean of all PFC regions (*t*s > 2.49, *p*s < 0.04), while there were no effects on male mice (*t*s < 0.59, *p*s > 0.80). Of note, orexin-positive fibres were detected in all PFC regions (Fig. 3C).

### Experiment 3

#### Spontaneous alternation in the Y-maze

**Fig. 4:**
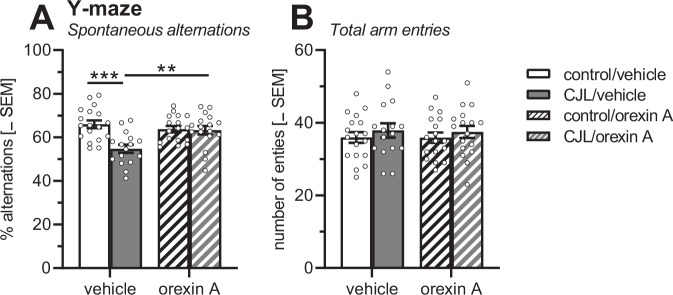
Effect of CJL and nasal orexin A treatment on spontaneous alternations and arm entries in the Y-maze. **A** CJL impaired the percentage of spontaneous alternations which was rescued by treatment with orexin A. **B** Both CJL and orexin A did not have effects on the total arm entries. ***p < 0.001, **p < 0.01, post-hoc comparison (as indicated) after significant effects in ANOVA. The dots represent the individual measures. CJL simulated chronic jet lag, control standard light-dark cycle.

Figure 4 depicts percent alternation and total arm visits of the mice in the Y-maze. Data were analysed with a multi-factorial ANOVA using sex (female/male), cycle condition (control/CJL), and treatment (vehicle, orexin A) as between-subject factors. Since no main effect of sex or interactions of sex with the other factors were found (F < 1.40; *p* > 0.24), the data of female and male mice were pooled. The ANOVA of the pooled data revealed a main effect of cycle condition (*F*(1,64) = 11.00; *p* = 0.002) and interaction of cycle condition with treatment (*F*(1,64) = 9.41; *p* = 0.003; Fig. 4A). Post-hoc comparisons showed that CJL impaired the percentage of alternations in vehicle-treated mice (*t* = 4.51, *p* < 0.0001) but not in orexin A-treated mice (*t* = 0.18, *p* = 0.86). CJL-exposed orexin A-treated mice had significantly more alternations than CJL-exposed vehicle-treated mice (*t* = 3.37, *p* = 0.001). Neither CJL nor orexin A treatment had any effects on the total number of arm entries in the Y-maze (*F*s < 1.17, *p*s > 0.28; Fig. 4B).

Separate analyses of female and male mice further are shown in the Supplements (Figs. S3, S4). The CJL effect was very similar in female and male mice, while the orexin A effect was more robust in females.

#### ASST performance

The performance of the mice in the ASST was analysed with multi-factorial ANOVAs using sex (female/male), cycle condition (control/CJL), and treatment (vehicle/orexin A) as between-subject factors and ASST phase (SD/CD/Rev1/IDS/Rev2/EDS/Rev3) as within-subject factor. Six mice failed to complete all ASST phases and were excluded from the final analyses (CJL/orexin A: *n* = 2 males; control/vehicle: *n* = 2 females; control/orexin A: *n* = 1 female, 1 male).

**Fig. 5:**
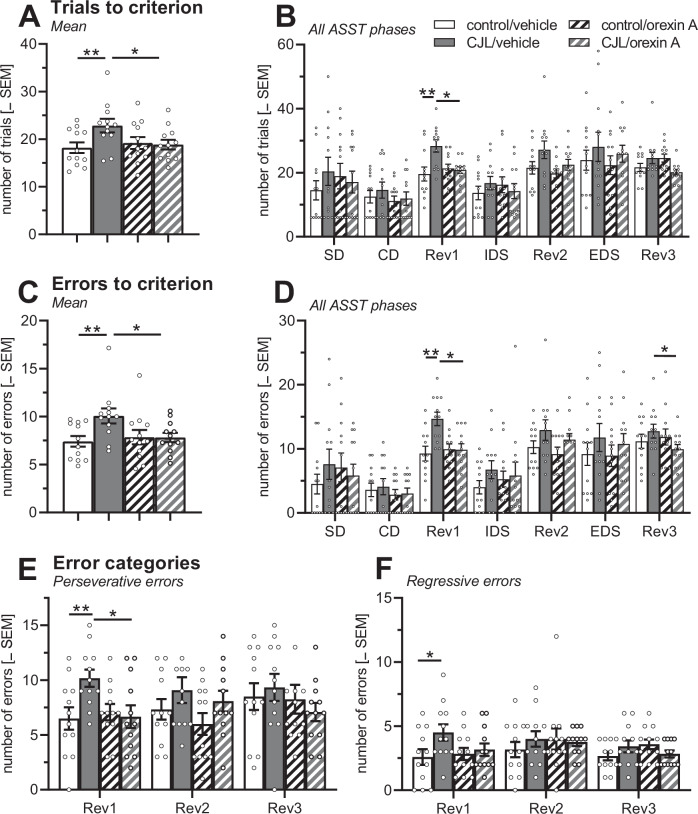
Effects of nasal administration of orexin A on impaired ASST performance after simulated CJL. **A** CJL increases the mean number of trials to criterion in vehicle-treated mice but not in orexin A-treated mice. **B** These effects were most pronounced in the Rev1 phase of ASST. **C** Very similar effects were observed on the mean number of errors to criterion. **D** These effects were most pronounced in the Rev1 and Rev3 phases. **E** Perseverative and **F** regressive errors were both increased in the Rev1 phase. **p < 0.01, *p < 0.05, post-hoc comparison (as indicated) after significant effects in ANOVA. The dots represent the individual measures. ASST attentional set shifting task, CD compound discrimination, CJL chronic jet lag, EDS extradimensional shift, IDS intradimensional shift, Rev1-3 reversal 1-3, control standard light-dark cycle.

A multi-factorial ANOVA revealed no main effects of sex (F < 1.64; *p* > 0.21) or interactions of sex with other factors (F < 0.81; *p* > 0.38) for trials to criterion (Fig. 5A, B), errors to criterion (Fig. 5C, D), and the errors types (Fig. 5E, F). Therefore, data from both sexes were pooled for further analysis, but data for female and male mice are also separately shown in the figures.

Regarding the trials and errors to criterion, no main effects of cycle conditions (Fs < 4.03, *p* > 0.05) and treatment (Fs < 1.94, *p* > 0.16) were found. However, there was an interaction between cycle condition and treatment (Fs > 4.25, *ps* = 0.045). Post-hoc comparisons showed that CJL increased the number of trials and errors in vehicle-treated mice (*t*s > 2.71, *p*s < 0.001) but not in orexin A-treated mice (*t*s < 0.44, *p*s > 0.66). In addition, there was a significant reduction of the number of trials in CJL-exposed mice after nasal orexin A administration (*t*s > 2.34, *p*s < 0.02). These effects were most pronounced in the Rev1 phase (*t*s > 3.00, *p*s < 0.008). In this phase, the analyses also revealed the effects of CJL on perseverative and regressive errors (*t*s > 2.84, *p*s < 0.01), as well as of orexin A on the CJL-induced increase of perseverative errors (*t* = 2.69, *p* = 0.02).

Separate analyses of female and male mice are further shown in the supplementary material (Figs. S5, S6). The described effects were very similar in female and male mice.

## Discussion

The aim of the present study was to investigate both the role and the potential of the orexin system in the presumably impairing effects of simulated CJL on executive functions in laboratory mice. The present data demonstrate that simulated CJL robustly impairs cognitive flexibility. In addition, simulated CJL decreased the activity of the orexin system and different subregions of the prefrontal cortex, a brain area critical for cognitive flexibility, at the time point when behavioural experiments started. Based on these findings, an interventional experiment was performed which demonstrated that nasal administration of orexin A rescued impaired cognitive flexibility and working memory after simulated CJL. Of note, the observed changes in the orexin system and the prefrontal cortex were only observed in female mice while there were no sex differences in the behavioural experiments. However, the behavioural changes were usually more pronounced in female mice.

In the present study, CJL was simulated to model cognitive symptoms of CRSD. Specifically, CRSD caused by jet lag and/or shift work was modelled [33]. Although occasional jet lag after long-distance travel is usually a problem that resolves itself, CJL in humans who travel frequently can cause a range of symptoms, including cognitive impairments [2, 3]. Additionally, between 10 and 15% of all workers are affected by shift work [33, 34]. Particularly, around one-third of shift workers suffer from shift work disorder [35], which is associated with cognitive impairments [36–38] and an increased risk of dementia [39]. Many of the described symptoms of CRSD are also observed in laboratory mice after simulated CJL and other forms of circadian desynchronization. Decreased motivation, increased anxiety, depression-like behaviours, reduced recognition memory and impaired spatial learning [7, 9, 40–43], but also molecular and physiological changes in different brain regions [44, 45], including the prefrontal cortex [7–10], and even increased mortality [46] have been observed. Of note, circadian desynchronization induced by transferring the mice to a 10:10 h light-dark cycle impairs cognitive flexibility, measured by reversal learning in the Morris water maze [7]. In humans, detrimental effects of CJL on executive functions such as cognitive flexibility or working memory have been demonstrated [36, 38], while knowledge of this in laboratory mice is limited.

The present study has shown that both cognitive flexibility and working memory are impaired in mice after simulated CJL. Our study demonstrates this in two independent experiments with different protocols of simulated CJL conducted by two different experimenters. A week protocol of CJL (6 h shift every seventh day) had no effects on cognitive flexibility after 1-month (4 shifts) but induced a significant impairment after 2 months (8 shifts). Therefore, a more severe protocol of CJL (8 h shift every fifth day) was used for the next experiments. With this protocol, impaired cognitive flexibility and working memory were observed after 4 weeks (6 shifts). Together, these results strongly support the findings in humans mentioned above and suggest that simulated CJL in mice could be used to study potential causes and treatments of CJL-induced cognitive impairment. It should be noted that home cage monitoring of mice’s spontaneous activity and/or sleep is necessary to demonstrate the non-cognitive symptoms of CRSD such as excessive sleepiness and lack of daytime alertness.

Working memory was only tested once in the present study, at the beginning of the light phase after the last shift. While we observed a robust deficit at this time point, it is not clear how long this deficit lasts, i.e., how long the effects of simulated CJL on working memory are. In contrast, the ASST lasts four days, and except for the first day, we observed deficits in at least one of the two ASST experiments on each of the following days of the ASST, suggesting that the effects of CJL on cognitive flexibility persist for at least four days. The absence of CJL-induced effects on the first day of ASST (including the simple discrimination phase) suggests that basal discrimination learning is not affected by CJL. All other phases of the ASST require cognitive flexibility, i.e., reversal learning or attentional shifts, and both of these processes appeared to be impaired in at least one of the two ASST experiments. It should be noted that the individual ASST phases show great variability and that the statistical power of single-phase analyses is not very high due to our sample sizes.

The orexin system is crucial for the regulation of the sleep-wake cycle [47, 48]; hence, it is reasonable to assume that this system is involved in CJL-induced symptoms [49, 50]. However, to the best of our knowledge, no published studies have shown that CJL and/or CRSD are associated with changes in the orexin system. At the time point of the start of the behavioural tests, the present study shows decreased activity of the orexin system after simulated CJL, a mouse model of CRSD. Surprisingly, this reduced activity was only observed in female mice, although the behavioural effects of CJL were very similar in both sexes. The same is true for neural activity in the prefrontal cortex, a crucial brain region for executive functions that is innervated by the orexin system [51]. Of note, c-Fos expression, which we used as a marker of neuronal activity, was only measured at one-time point. This time point fits to the Y-maze test and the first day of the ASST but does not allow any statement about potential neuronal activity changes on the following days. The absence of these changes in male mice is unclear; however, it is possible that changes, if any, can simply not be detected with the c-Fos approach. An attempt was made to measure orexin levels in the PFC in the present study, however, the ELISA used also measured orexin levels in orexin-deficient mice, so we discontinued these measurements. Future experiments should include more sensitive methods to measure the activity of the orexin system and the orexin levels of the PFC. Measuring more time points will also help to evaluate whether the observed changes in c-Fos expression are the consequence of shifted peak times, as the internal clock of the mice is shifted.

As already mentioned, the PFC is crucial for executive functions. Specifically, the medial PFC is important for working memory and extradimensional set shifting, while the orbitofrontal cortex is important for reversal learning and the cingulate cortex for intradimensional set shifting [52–54]. We analysed c-Fos expression in all these subregions of the PFC and observed similar effects of CJL in all subregions, at least in female mice. This implies that the entire PFC and not only specific subregions are affected by CJL and therefore other cognitive abilities mediated by the PFC should also be affected.

The observed decrease in neuronal activity in the PFC could be a consequence of the impaired activity of the orexin system. This reduced PFC activity could in turn cause behavioural deficits in cognitive flexibility and working memory tests. If this is the case, a pharmacological intervention that replaces the missing orexin in the PFC should rescue the observed behavioural deficits. A relatively simple approach to administer orexin to the brain is via the nasal route [55], which has been shown to increase orexin levels in the brain [56, 57] and activate the PFC [58]. Indeed, after nasal administration of orexin, we observed a complete rescue of impaired cognitive flexibility and working memory after simulated CJL. This rescue effect of nasal orexin was also observed in male mice, although we could not detect impaired activity in the orexin system and the PFC in them. Of course, CJL could not only reduce neuronal activity in the PFC but also induce the above-mentioned physiological and molecular changes in the PFC. These changes may be more pronounced in male mice (instead of increased neuronal activity) and may also respond to nasal administration of orexin.

In the present study, a dose of orexin was used that is comparable to doses used to rescue deficits of aged rats in the ASST [30]. The authors of this study also show that a single nasal administration has a relatively quick effect on transmitter concentrations and/or neuronal activity in different brain regions and that this effect lasts for about one hour. In another study in rats, radioactive orexin was used and still found in different brain areas 2 h after nasal administration [56]. To our knowledge, there are no comparable studies in mice, but the present data suggest that nasally administrated orexin affects brain activity and/or physiology for the duration of the daily ASST tests (approximately 2–3 h) and can thereby rescue CJL-induced cognitive deficits. Since we tested only one dose of orexin, we cannot make any statement about the dose-dependency of the observed effect. Furthermore, we performed all our experiments at the beginning of the light phase, i.e., the inactive phase of mice, and any effects observed in the present study could be different at other time points of the light/dark cycle, e.g., at the end of the light phase or in the dark phase. We hypothesize that orexin administration at the beginning of the inactive period primarily increases the arousal of the mice and thus also cognitive performance. However, increased arousal can also act as a zeitgeber and thus help to (re)synchronize circadian desynchronization. Additionally, orexin also directly affects neurons that are important for executive functions, e.g., parvalbumin-positive interneurons in the PFC, and could thereby rescue cognitive deficits [30, 59].

Overall, the present findings show that the orexin system is involved in the cognitive impairments observed after simulated CJL and that nasal orexin administration rescues these impairments. Although there were some sex differences – as frequently observed in orexin research [21, 29, 60] – in the effect of simulated CJL on neural activity, nasal orexin administration had similar effects in female and male mice. These data suggest that nasal orexin administration is a potential treatment option for symptoms of CRSD. Intranasal orexin administration has been tested in narcoleptic patients who have very low or absent orexin levels in the brain [28] and could alleviate several symptoms in these patients such as olfactory dysfunction, attentional deficits and sleep abnormalities without significant side effects [61–63]. Therefore, nasal orexin administration should be tested in CRSD patients.

## Supplementary information

Supplementary information
