# Supplementary material for: Orexinergic modulation of chronic jet lag-induced deficits in mouse cognitive flexibility
Source: Neuropsychopharmacology. 2024 Oct 30;50(5):762–71. doi: 10.1038/s41386-024-02017-8 (PMC11914050; doi:10.1038/s41386-024-02017-8)
Supplement: Supplementary file 1 — Supplementary information [file 41386_2024_2017_MOESM1_ESM.pdf]

Supplementary MATERIALS AND METHODS

Attentional Set Shifting Task (ASST)

Custom-made boxes (41 cm × 22 cm × 24 cm) were used, subdivided into a waiting and a choice area, latter consisting of two compartments that were separated by each other by a fixed transparent wall. Further, there were transparent sliding doors between the waiting area and the choice area. A bowl (diameter: 5 cm, height: 3 cm) with water was placed in the waiting area, two identical bowls were placed in the two compartments of the choice area. During the ASST pretraining phases, the bowls in the choice area were filled with bedding material. However, during the critical phases of the ASST, these bowls were filled with digging medium as visual-tactile cues. Further, odorants, dropped on a filter paper that was fixed on the outside of the bowls were used as olfactory cues. As digging media wooden green pearls, white grains, and sand balls, each either of small or big and size, were used. For the olfactory cues, citral, eucalyptol, S-(+)-carvone, R-(-)-carvone, valeric acid, and 2-phenylethanol (all from Sigma-Aldrich, St. Louis, Missouri, USA) were diluted 1:20 in paraffin oil. Half of a Choco Rice (ca. 20 mg, Nordgetreide GmbH & Co. KG, Lübeck, Germany) was used as a reward.

Habituation Phase: The mice were handled by the experimenter daily for one week. At the fourth day, food restriction (ca. 2.5 g/day per animal) started until the end of the ASST, with the goal to reduce basal body weight by 5-10%. On the same day, mice were habituated to the bowls and the reward by placing them overnight in the home cage. One day later, all mice of a cage were habituated to the ASST setup by putting them together in the boxes for ca. 40 min. After a break of two days, each mouse then was systematically trained to dig and retrieve the reward from the bowls, filled with bedding material, for 12 trials (4 trials with rewards on the top of one bowl, four trials with the reward slightly covered by bedding material, four rewards with reward on the bottom of the bowl). For experiment 3, mice were also habituated to nasal application of saline on the last two days of the habituation phase.

Testing Phase: Directly after the habituation phase, the actual ASST was performed. During four consecutive days, the bowls were presented with the visual-tactile and/or olfactory cues described above. Only one of the two bowls placed in the compartments of the choice area contained a reward. The mice had to learn which of the cues was associated with the reward-baited bowl in order to retrieve the reward. The position of the bowl with the reward was pseudorandomized from trial to trial. To prevent the mouse from detecting the reward by its odor, the bottoms of both bowls were sprinkled with the powder of Choco Rice, i.e., both bowls smelled of but only one bowl contained the reward. Half of the mice started the first testing phase (SD: simple discrimination) with olfactory cues (odorants) as the relevant dimension and

the other half with visual-tactile cues (digging medium) as the relevant, i.e., reward-associated dimension. At the beginning of each trial, a mouse was placed in the waiting area for 30 seconds. Then, according to a pseudorandomized protocol, the two doors of the choice area were opened (correct door first, wrong door first, both doors simultaneously), so that the mouse could enter the compartments and were able to decide for one of the two bowls. The decision of the mouse was defined by digging or moving the material in the bowl with the nose or paws. After the mouse's decision for one bowl, the door of the other compartment was closed. If this other compartment's bowl contained the reward, this bowl was removed by the experimenter. This removal should serve as an additional cue for the mouse that it made a wrong decision. If the choice was correct, the mice were allowed to eat the reward completely before it was gently guided back to the waiting area for the beginning of the next trial. There were two ways for the mouse to reach the criterion for successful completion of an ASST phase and thereby to move on to the next phase. One option was to make six consecutive correct decisions. Alternatively, if an error was made after five correct consecutive decisions, the mouse could correct this by making a correct decision immediately after this error. The ASST phases SD, CD, and IDS (see below) started with four so-called free trials. In contrast to the subsequent runs, both doors remained open throughout a trial and the mouse could correct a wrong decision. The free trials were not taken into account for the criterion for successful completion of a phase. If a mouse did not dig any of the bowls for more than 5 min, the experiment was temporarily paused and resumed when the mice expressed exploratory behaviour again.
In detail, the testing phases of the ASST consisted of the following phases:

- Simple discrimination (SD): Two different exemplars of one stimulus dimension , i.e., either olfactory cues (odorants) or visual-tactile cues (digging medium) were presented. If odorants were used as the first stimulus dimension, bowls were filled with bedding material. <u>Example (see table below):</u> The bowls with the large green pearls were rewarded, the bowls with small green pearls not.
- Compound discrimination (CD): Two exemplars of an additional but irrelevant stimulus dimension (digging medium, if odorant was the starting stimulus dimension, and odorant, if digging medium was the starting stimulus dimension) were introduced. CD was followed by the first reversal phase (Rev1), in which the contingency of the relevant cues changed. <u>Example (see table below):</u> In CD, the bowls with the large green pearls were rewarded, regardless of the odor (eucalyptol or 2-phenylethanol). The bowls with the small pearls were not rewarded, regardless of the odor (eucalyptol or 2-phenylethanol). In Rev1, the bowls with small green pearls were rewarded (regardless of odor), the bowls with large green pearls not (regardless of odor).
- Intradimensional shift (IDS): New exemplars of both stimulus dimensions were presented. One of the new exemplars of the previously relevant dimension predicted the

reward. The IDS phase was followed by the second reversal phase (Rev2). Example (see table below): In IDS, the bowls with the large white grains were rewarded, regardless of the odor (citral or R-carvone). The bowls with the small white grains were not rewarded, regardless of the odor (citral or R-carvone). In Rev2, the bowls with small white grains were rewarded (regardless of odor), large white grains not (regardless of odor).

- Extradimensional shift (EDS): New exemplars for both stimulus dimensions were presented. Of note, one of the new exemplars of the previously irrelevant dimension predicted the reward. EDS was followed by a third reversal phase (Rev3). Example (see table below): In EDS, the bowls with the valeric acid were rewarded, regardless of the medium (large or small sand balls). The bowls with the S-carvone were not rewarded, regardless of the odor (large or small sand balls). In Rev1, the bowls with S-carvone were rewarded (regardless of medium), the bowls with valeric acid not (regardless of medium).

Table with an example:

	**relevant dimension**	rewarded	non-rewarded
**SD**	**medium**	**M5 (large green pearls)**	M6 (small green pearls)
**CD**	**medium**	**M5 (large green pearls)** + O3 (eucalyptol)	M6 (small green pearls) + O3 (eucalyptol)
		**M5 (large green pearls)** + O4 (2-phenylethanol)	M6 (small green pearls) + O4 (2-phenylethanol)
**Rev1**	**medium**	**M6 (small green pearls)** + O3 (eucalyptol)	M5 (large green pearls) + O3 (eucalyptol)
		**M6 (small green pearls)** + O4 (2-phenylethanol)	M5 (large green pearls) + O4 (2-phenylethanol)
**IDS**	**medium**	**M3 (large white grains)** + O5 (citral)	M4 (small white grains) + O5 (citral)
		**M3 (large white grains)** + O6 (R-carvone)	M4 (small white grains) + O6 (R-carvone)
**Rev2**	**medium**	**M4 (small white grains)** + O5 (citral)	M3 (large white grains) + O5 (citral)
		**M4 (small white grains)** + O6 (R-carvone)	M3 (large white grains) + O6 (R-carvone)
**EDS**	**odor**	M1 (large sand balls) +	M1 (large sand balls) +

		**O1 (valeric acid)**	O2 (S-carvone)
		M2 (small sand balls) + **O1 (valeric acid)**	M2 (small sand balls) + O2 (S-carvone)
**Rev3**	**odor**	M1 (large sand balls) + **O2 (S-carvone)**	M1 (large sand balls) + O1 (valeric acid)
		M2 (small sand balls) + **O2 (S-carvone)**	M2 (small sand balls) + O1 (valeric acid)

Software “ForAnne” to count cFos-positive neurons

The software was written by Anne Rakowsky and Jonas Mucke in the Python programming language and has a graphical user interface that allows the user to read the microscopic images and mark certain areas of the brain. There are then various processing steps that are very similar to those in professional software: (1) The image is converted to greyscale. (2) Use of various image filters: Gaussian filter for noise reduction, sharpening filter for sharpening the edges. (3) Conversion to a black and white image. (4) Using an island detection algorithm to recognise and count the number of disjoint objects in the image.

The analysis of this software was validated by comparison with manual analysis and automatic cell count analysis using Leica LAS X software and ImageJ.

The software is available on request (markus.fendt@med.ovgu.de).

Supplementary RESULTS

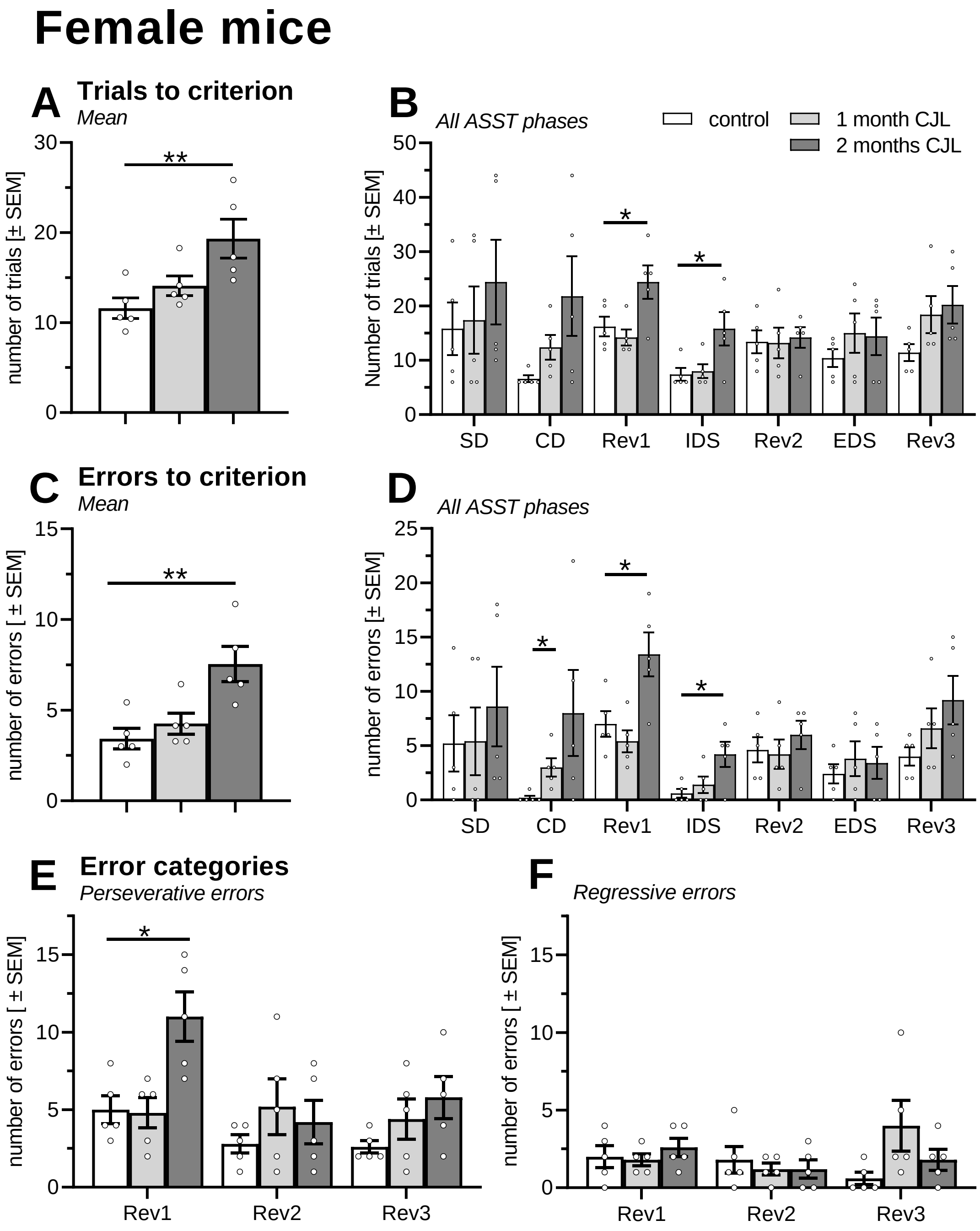

Fig. S1. ASST performance after one or two months of simulated CJL (female mice). A Two but not one month of CJL increase the mean number of trials to criterion compared with standard light/dark cycle. **B** This effect was most pronounced in the CD, Rev1, IDS and Rev3 phase of ASST. **C** Very similar effects were observed on the mean number of errors to criterion. **D** This effect were most pronounced in the CD, Rev1 and IDS phase. **E** Perseverative and **F** regressive errors were not affected on a statistical level. ** $p < 0.01$, * $p < 0.05$, post-hoc comparisons (as indicated) after significant effects in ANOVA. The dots represent the individual measures. Abbreviations: ASST, attentional set shifting task; CD, compound discrimination; CJL, chronic jet lag; EDS, extradimensional shift; IDS, intradimensional shift; Rev1-3, reversal 1-3; ST, standard light-dark cycle.

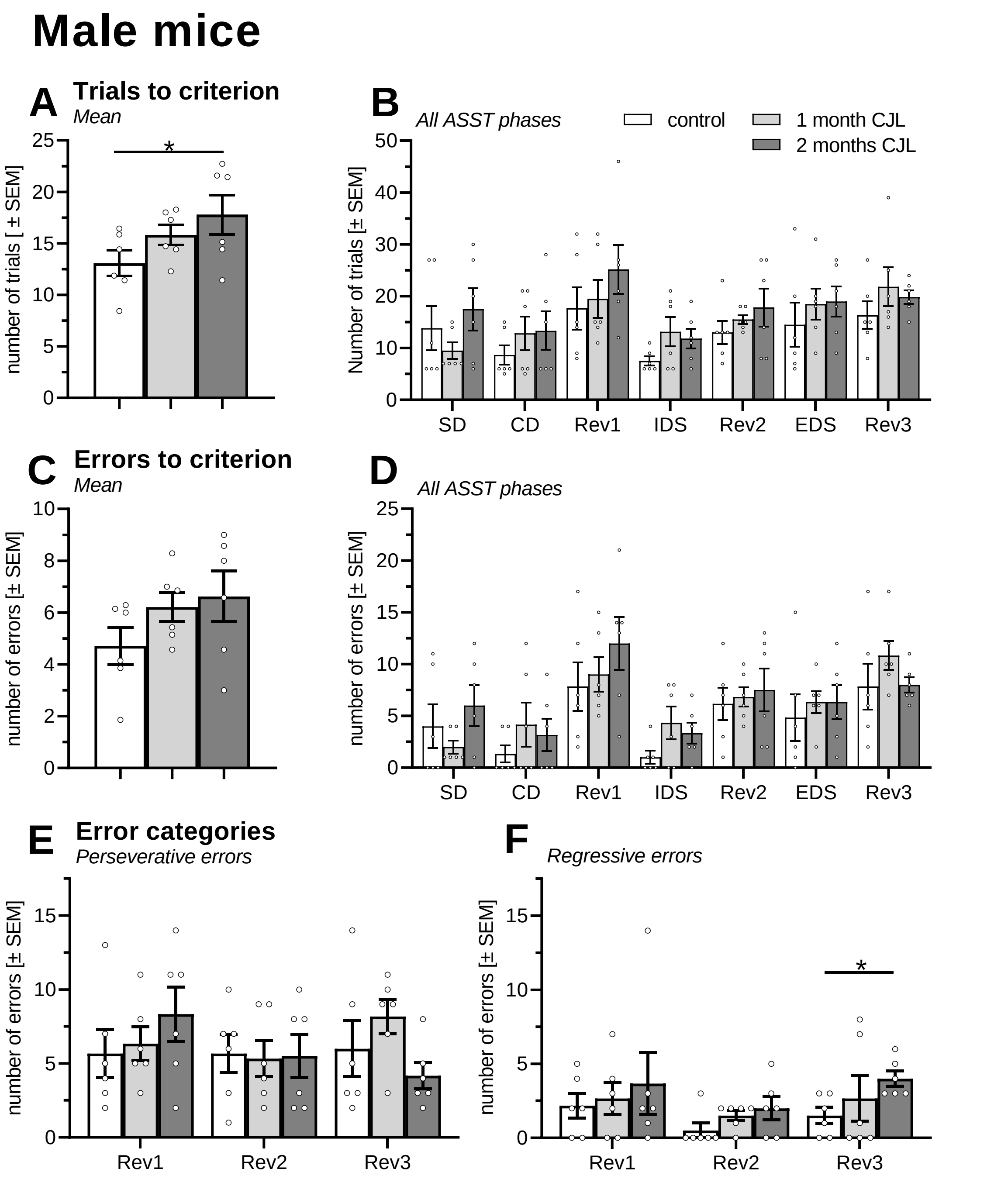

Fig. S2. ASST performance after one or two months of simulated CJL (male mice). A Two but not one month of CJL increase the mean number of trials to criterion compared with standard light/dark cycle. **B** This effect was most pronounced in the CD, Rev1, IDS and Rev3 phase of ASST. **C** Very similar effects were observed on the mean number of errors to criterion. **D** This effect were most pronounced in the CD, Rev1 and IDS phase. **E** Perseverative and **F** regressive errors were not affected on a statistical level. * $p < 0.05$, post-hoc comparisons (as indicated) after significant effects in ANOVA. The dots represent the individual measures. Abbreviations: ASST, attentional set shifting task; CD, compound discrimination; CJL, chronic jet lag; EDS, extradimensional shift; IDS, intradimensional shift; Rev1-3, reversal 1-3; ST, standard light-dark cycle.

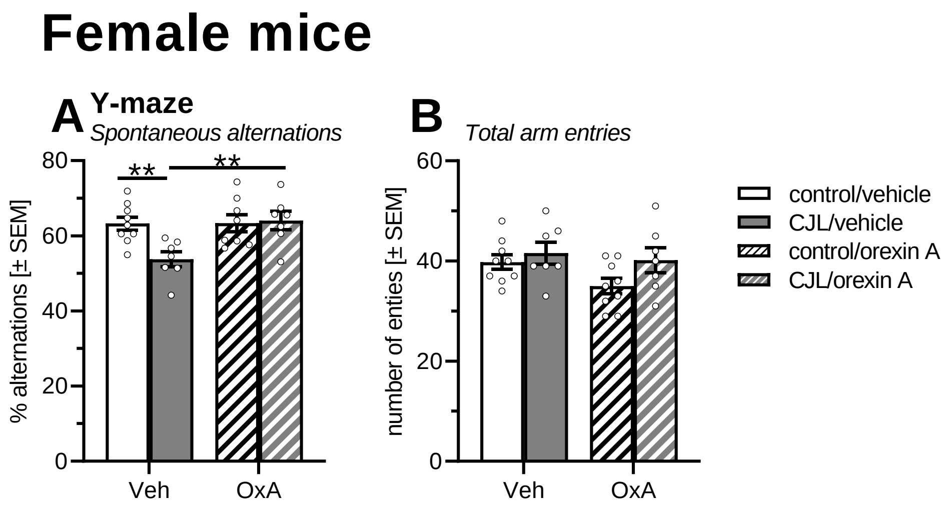

Fig. S3. Effect of CJL and OxA treatment on spontaneous alternations and arm entries in the Y-maze (female mice). A CJL impaired the percentage of spontaneous alternations which was rescued by treatment with OxA. **B** Both CJL and OxA did not had effects on the total arm entries. ** $p < 0.01$, post-hoc comparison (as indicated) after significant effects in ANOVA. The dots represent the individual measures. Abbreviations: CJL, simulated chronic jet lag; OxA, orexin A; ST, standard light-dark cycle; Veh, vehicle.

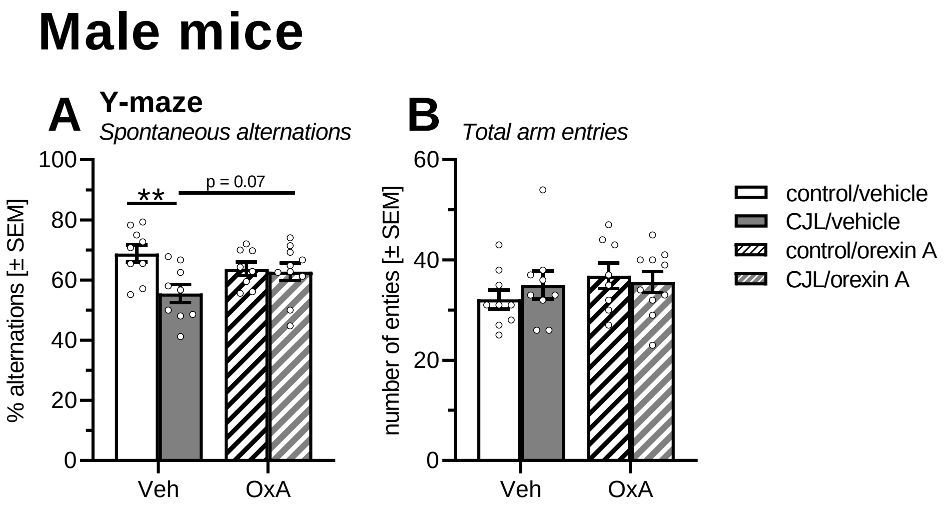

Fig. S4. Effect of CJL and OxA treatment on spontaneous alternations and arm entries in the Y-maze (male mice). A CJL impaired the percentage of spontaneous alternations which was rescued by treatment with OxA. **B** Both CJL and OxA did not had effects on the total arm entries. ** $p < 0.01$, post-hoc comparison (as indicated) after significant effects in ANOVA. The dots represent the individual measures. Abbreviations: CJL, simulated chronic jet lag; OxA, orexin A; ST, standard light-dark cycle; Veh, vehicle.

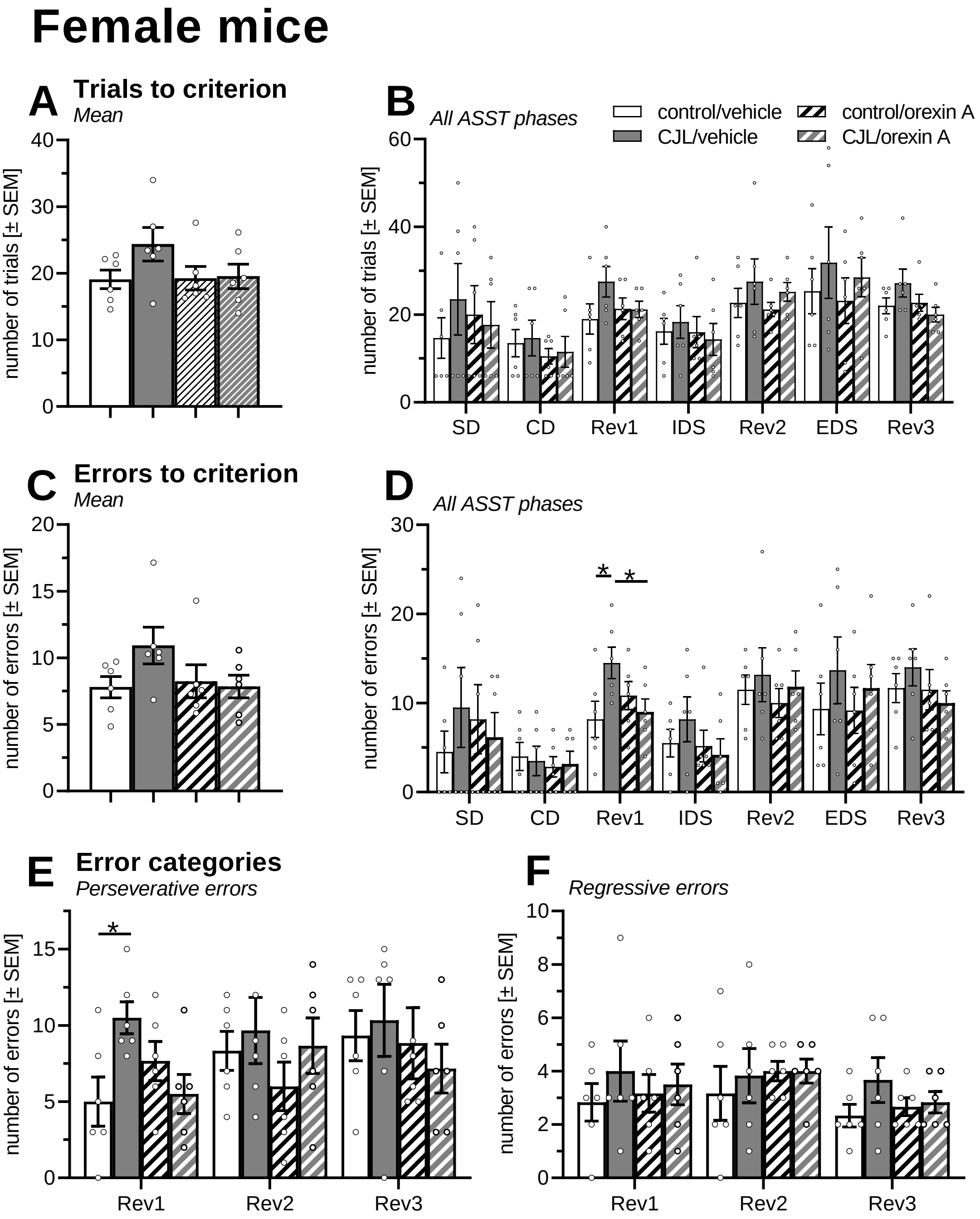

Fig. S5. Effects of oral administration of orexin A on impaired ASST performance after simulated CJL (female mice). A CJL increases the mean number of trials to criterion in vehicle-treated mice but not in orexin A-treated mice. **B** These effects were most pronounced in the Rev1 phase of ASST. **C** Very similar effects were observed on the mean number of errors to criterion. **D** These effects were most pronounced in the Rev1 and Rev3 phase. **E** Perseverative and **F** regressive errors were both increased in the Rev1 phase. * $p < 0.05$, post-hoc comparison (as indicated) after significant effects in ANOVA. The dots represent the individual measures. Abbreviations: ASST, attentional set shifting task; CD, compound discrimination; CJL, chronic jet lag; EDS, extradimensional shift; IDS, intradimensional shift; Rev1-3, reversal 1-3; ST, standard light-dark cycle; Veh, vehicle.

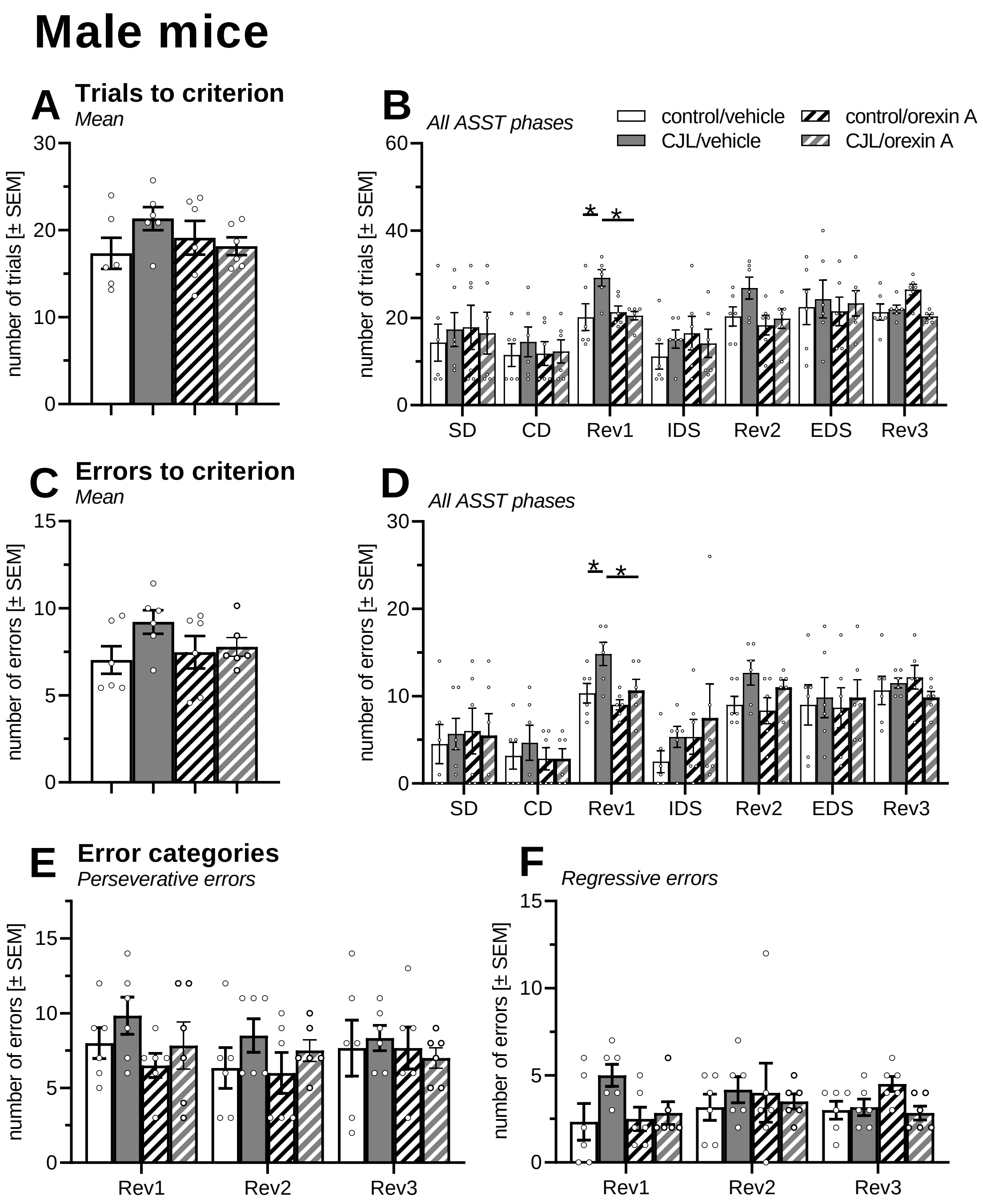

Fig. S6. Effects of oral administration of orexin A on impaired ASST performance after simulated CJL (male mice). A CJL increases the mean number of trials to criterion in vehicle-treated mice but not in orexin A-treated mice. **B** These effects were most pronounced in the Rev1 phase of ASST. **C** Very similar effects were observed on the mean number of errors to criterion. **D** These effects were most pronounced in the Rev1 and Rev3 phase. **E** Perseverative and **F** regressive errors were both increased in the Rev1 phase. * $p < 0.05$, post-hoc comparison (as indicated) after significant effects in ANOVA. The dots represent the individual measures. Abbreviations: ASST, attentional set shifting task; CD, compound discrimination; CJL, chronic jet lag; EDS, extradimensional shift; IDS, intradimensional shift; Rev1-3, reversal 1-3; ST, standard light-dark cycle; Veh, vehicle.
